# Heart-Strain and School Athletics

**Published:** 1905-04-08

**Authors:** 


### HEART-STRAIN AND SCHOOL ATHLETICS.

The common field sports of public school life are not without their opponents both from the "flannelled fool, muddied oaf" point of view, and also on the score of the physical damage likely to accrue from over-exertion. It is comforting therefore to find a doctor who has had experience in connection with one of the largest public schools expressing himself in optimistic terms as to the physical aspect of the matter. Dr. Arthur Lambert,[1] speaking from experience at Harrow, says: "I am led to doubt whether the heart of the truly healthy boy ever breaks down as the result of athletics as practised in our great public schools. The schoolboy heart breaks down as the result of exertion, not because the strain is too severe for the hearts of boys of that age or physical development, but because the individual boy possesses some cardiac insufficiency, either primary or dependent upon some condition such as anæmia or recent influenza."

Dealing with some cases of heart-strain in growing boys which have come under his notice, Dr. Lambert discusses the relation of repeated physiological distension of the heart with pathological dilatation; the question, that is to say, whether a heart in the first instance sound in all particulars may be reduced to a state of pathological dilatation by the frequent repetition of the distension incidental to ordinary athletics. He is of opinion that the answer must be negative, and that the occurrence of pathological dilatation is evidence of an inherent myocardial fault, for the condition is rare.

The class of case to which the author alludes is illustrated by the three following, taken from his series. G. M., a good boxer, aged 17, became very breathless during a round, and by the end was exceedingly embarrassed in his breathing. On examination it was found that his heart was much hypertrophied, and the impulse heaving; the apex-beat was in the sixth space a quarter of an inch outside the nipple line; the pulse-tension was low and the rate 148.

Again, a boy of 13, while bicycling up an incline which he had often mounted before, became suddenly faint. On examination his heart was found to be dilated. He recovered, to all appearance, completely, except that in the recumbent position his pulse became intermittent. He was allowed to resume his exercise gradually, but in the following year the dilatation recurred, and was associated with a mitral systolic murmur. He went to Nauheim, where the murmur disappeared, and he returned recovered, only to break down again, and finally had to forswear violent exercises altogether.

In a third very similar case a boy of 15 became suddenly faint while wheeling a heavy barrow, and was found to have a dilated heart; he also recovered, but subsequently relapsed again while bowling, and was compelled to give up games.

Dr. Lambert has seen the same tendency to recurrence of the symptoms of dilatation as a result of direct violence, and, as he believes, of mental emotion. For instance, a healthy boy, while playing football, stumbled and rolled on his back, when a playfellow came heavily on his præcordia with one knee. The boy became collapsed and showed marked cardiac dilatation. He recovered for the time, but later, on attempting to resume violent exercise, relapsed and had to take to his bed.

Again, a boy of 15 who came under notice on account of cardiac dilatation during a game of football had the following history. He was in perfect health until two years before when he accidentally shot another boy dead. At that time he fainted and showed signs of cardiac dilatation, and had had one other similar attack in the interval.

As to the prognosis, the author considers it should always be a guarded one, in view of the likelihood of recurrence. We cannot do better than quote his own expressions on the matter. "I believe that pathological dilatation of the heart of the growing boy from strain, however short its duration, and however complete its apparent cure, leaves its indelible mark upon the mechanism of the heart, and that its effects have to be regarded as an existing factor . . . sufficient perhaps to determine the failure of the heart under an anæsthetic, or to decide whether in some acute illness the resultant of the forces acting in and on the patient shall take the direction of recovery or death."

Dr. Lambert specifically excludes rowing from the athletics of whose effects he has had experience. Now a rowing-race is perhaps the best example of sustained high pressure that can be found, yet the number of old University oarsmen who have lived to a ripe old age on the bench and in other departments does not support a condemnation of the practice. Most people we think will agree with Dr. Lambert that a sound boy may undergo without damage any calls which public-school athletics may make upon his heart, provided always that a fair preparation in the way of training precedes any great effort. The real danger in these matters attends those whom a sudden emergency persuades to embark upon a great trial of endurance without any, or without an adequate, preliminary period of training.

[1] Medical Chronicle, February 1905.

